# Suppression of aggregate and amyloid formation by a novel intrinsically disordered region in metazoan Hsp110 chaperones

**DOI:** 10.1016/j.jbc.2021.100567

**Published:** 2021-03-19

**Authors:** Unekwu M. Yakubu, Kevin A. Morano

**Affiliations:** 1 Department of Microbiology and Molecular Genetics, McGovern Medical School at UTHealth, Houston, Texas, USA; 2 MD Anderson UTHealth Graduate School at UTHealth, Houston, Texas, USA

**Keywords:** Hsp70, Hsp110, chaperone, Alzheimer’s disease, Parkinson’s disease, amyloid, protein aggregation, proteostasis, ALS, amyotrophic lateral sclerosis, APP, amyloid precursor protein, BSA, bovine serum albumin, CS, citrate synthase, Htt, Huntingtin, IDR, intrinsically disordered region, iMAC, immobilized affinity chromatography, NBD, nucleotide-binding domain, NEF, nucleotide exchange factor, PFF, preformed fibril, SBD, substrate-binding domain, TEM, transmission electron microscopy

## Abstract

Molecular chaperones maintain proteostasis by ensuring the proper folding of polypeptides. Loss of proteostasis has been linked to numerous neurodegenerative disorders including Alzheimer's, Parkinson's, and Huntington's disease. Hsp110 is related to the canonical Hsp70 class of protein-folding molecular chaperones and interacts with Hsp70 as a nucleotide exchange factor (NEF). In addition to its NEF activity, Hsp110 possesses an Hsp70-like substrate-binding domain (SBD) whose biological roles remain undefined. Previous work in *Drosophila melanogaster* has implicated the sole Hsp110 gene (Hsc70cb) in proteinopathic neurodegeneration. We hypothesize that in addition to its role as an Hsp70 NEF, *Drosophila* Hsp110 may function as a protective protein "holdase," preventing the aggregation of unfolded polypeptides *via* the SBD-β subdomain. We demonstrate for the first time that *Drosophila* Hsp110 effectively prevents aggregation of the model substrate citrate synthase. We also report the discovery of a redundant and heretofore unknown potent holdase capacity in a 138-amino-acid region of Hsp110 carboxyl terminal to both SBD-β and SBD-α (henceforth called the C-terminal extension). This sequence is highly conserved in metazoan Hsp110 genes, completely absent from fungal representatives, and is computationally predicted to contain an intrinsically disordered region (IDR). We demonstrate that this IDR sequence within the human Hsp110s, Apg-1 and Hsp105α, inhibits the formation of amyloid Aβ-42 and α-synuclein fibrils *in vitro* but cannot mediate fibril disassembly. Together these findings establish capacity for metazoan Hsp110 chaperones to suppress both general protein aggregation and amyloidogenesis, raising the possibility of exploitation of this IDR for therapeutic benefit.

Molecular chaperones support cellular protein homeostasis (proteostasis) by assisting in the proper folding and assembly of nascent polypeptide chains, regulating activity and interactions of mature proteins, and shepherding damaged or short-lived proteins to degradation pathways (1). Environmental stressors such as oxidation, heat shock, or starvation are especially detrimental to incompletely folded proteins that have a propensity to misfold and form aggregates. In a balanced proteostatic network, molecular chaperones such as Hsp70 and its cofactors prevent or reverse protein aggregation caused by partially or misfolded polypeptides (2, 3). However, with age and prolonged cellular damage, proteostasis becomes dysregulated leading to increased protein aggregation and resultant cytotoxicity (4, 5, 6, 7).

Numerous neurodegenerative disorders such as Alzheimer’s, Parkinson’s, and Huntington’s disease have been linked to the misfolding and aggregation of key proteins in neuronal and muscle cells (4, 8). The misfolding of amyloidogenic polypeptides leads to the formation of structured oligomers that act as seeding elements for other amyloid-prone proteins as well as nonpathogenic polypeptides (9, 10, 11). Over time these amorphous oligomers form higher-order energetically favorable and toxic amyloid fibrils, which are difficult for the cell to disassemble. These occlusive fibrils, and likely the oligomeric intermediates that precede them, incorporate partially folded proteins, titrate chaperones away from nascent polypeptides, and contribute to overall cellular toxicity that ultimately results in neuronal cell death (5). Understanding the molecular mechanisms behind amyloidogenesis and its contribution to neurodegeneration can result in the development of therapeutics that halt or even reverse the progression of these devastating diseases. This is especially important given rapidly aging populations around the world, as many of these diseases, most notably Alzheimer’s disease, primarily affect persons aged 65 and older (5, 12).

The Hsp70 nucleotide exchange factor Hsp110 specifically plays a role in preventing the aggregation of neurodegenerative disease-associated polypeptides as demonstrated *in vitro* and *in vivo* with the use of animal models (13, 14, 15, 16). The *Drosophila melanogaster* Hsp110 homolog Hsc70Cb, when overproduced in fly lines also expressing human Huntingtin (Htt) exon 1, prevents Htt aggregation and associated neurodegenerative phenotypes (17). Likewise, overexpression of Hsp110 mitigates protein aggregation and neurodegeneration in Parkinson’s and amyotrophic lateral sclerosis (ALS) mouse models (13, 18). Conversely, deletion of Hsp110 homologs in mice and *Drosophila* results in enhanced neurodegeneration resulting from increased amyloid formation of Alzheimer’s-linked Aβ_1-42_ and expanded polyglutamine protein, respectively (16, 19). Hsp110 is a member of the Hsp70 superfamily and as such shares the canonical Hsp70 architecture: an amino-terminal nucleotide-binding domain (NBD) followed by a short linker and a substrate-binding domain (SBD) comprising two subdomains termed SBD-β and SBD-α (Fig. 1*A*). SBD-β is a β-sandwich-containing putative substrate-binding site, while SBD-α is an α-helical sequence that, instead of clamping over the SBD-β region with associated peptide substrate as in all Hsp70 chaperones, binds to and stabilizes the Hsp110 NBD, promoting interaction with Hsp70 to form a stable heterodimer that facilitates nucleotide exchange (20, 21, 22, 23).

**Figure 1:**
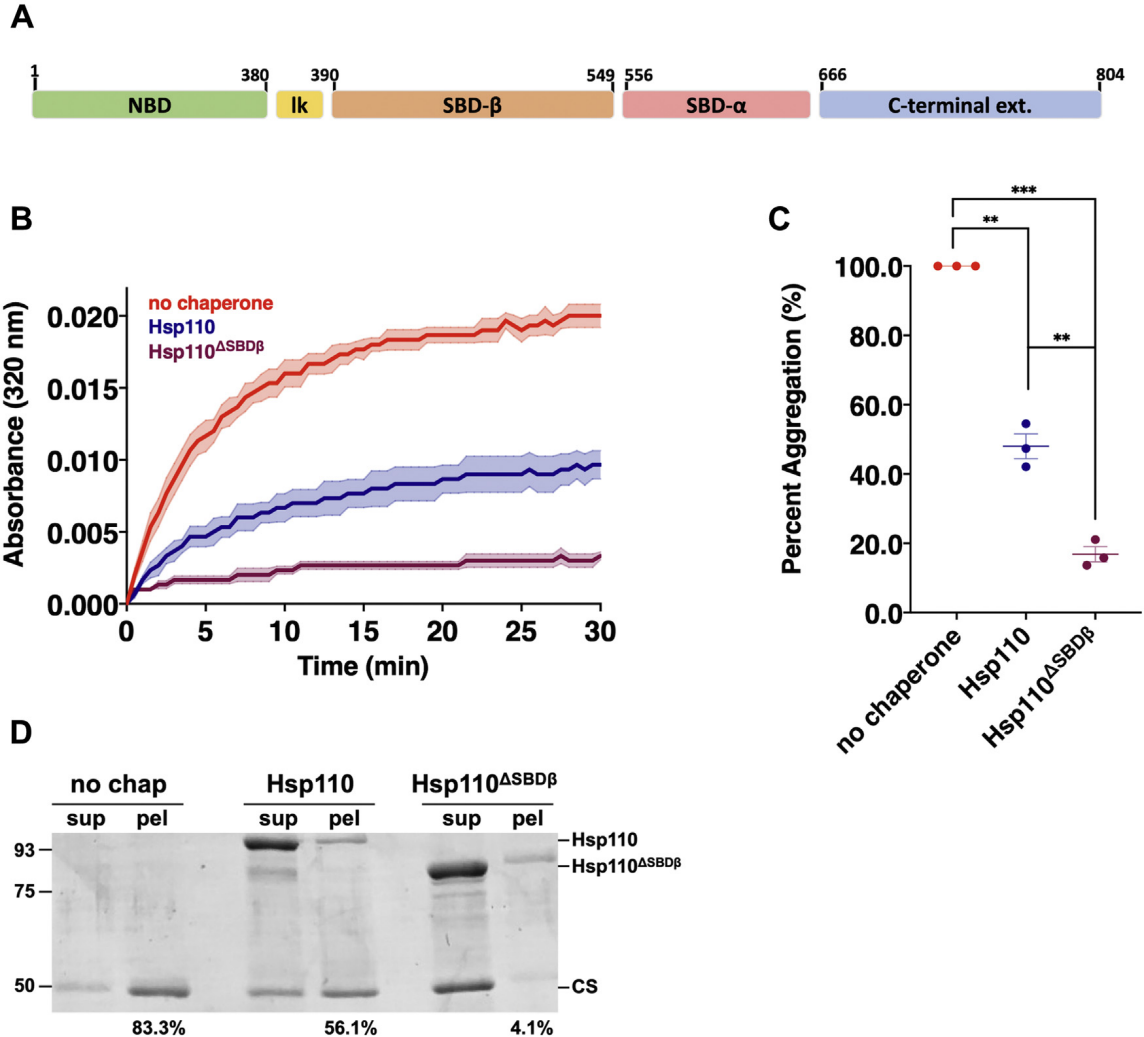
**The *Drosophila* Hsp110 substrate-binding domain, SBD-β, is expendable for aggregation suppression activity.***A*, schematic of Hsp110 domain architecture: nucleotide-binding domain (*green*) a.a. 1–380; linker (*yellow*) a.a. 381–389; substrate-binding domain β (*orange*) a.a. 390–549; substrate-binding domain α (*pink*) a.a. 556–665; C-terminal extension (*blue*) a.a. 666–804. *B*, in total, 200 nM denatured citrate synthase (CS) was incubated alone (no chaperone) or with 400 nM of respective chaperone: Hsp110, Hsp110^ΔSBDβ^ and light scattering measured at 320 nm, Bolded lines are the average of three replicates for each condition while the shaded region represents standard error of the mean (SEM). *C*, end point measurements of each condition were taken from (*B*) and divided by the no chaperone measurement within the respective replicate and converted to relative percentage. Group differences were analyzed using Welch’s *t-*test. ∗*p* = 0.05; ∗∗*p* = 0.005; ∗∗∗*p* = 0.0005; ∗∗∗∗*p* = 0.00005. *D*, following light scatter assay end point samples were separated into soluble (sup) and insoluble (pel) fractions by differential centrifugation. Numbers indicate per cent of pellet signal, as quantified using ImageJ, from Coomassie-stained SDS-PAGE gel relative to combined sup plus pel signals.

While the NEF activity of Hsp110 has been well characterized, the biological role of substrate binding by the chaperone remains elusive (22, 24). *In vitro* studies have demonstrated that Hsp110 is a potent suppressor of protein aggregation in the absence of Hsp70, and SBD-β appears to dictate peptide binding in the yeast homolog Sse1 (25, 26, 27, 28). Additionally, substrate binding by Sse1 is not required for the disaggregase activity exhibited by the Hsp40-Hsp70-Hsp110 triad (20). Moreover, Hsp110 appears to optimally function as an NEF at a 1:10 ratio with respect to Hsp70, significantly inhibiting Hsp70 activity at higher levels (14, 23, 29). This stoichiometry is inconsistent with the clear amyloid-suppressing effects observed upon overexpression in animal models of human neurodegenerative disease. Given the compelling protection from Htt-induced neurotoxicity provided by Hsp110 overexpression in flies, we interrogated protein aggregation inhibition by fly Hsp110 through mutational analysis and revealed a previously undiscovered second substrate-binding site in the extreme carboxyl terminus. This region, henceforth referred to as the C-terminal extension, is absent in yeast Sse1 but conserved in Hsp110 genes from metazoans. We demonstrate that an intrinsically disordered region (IDR) within the C-terminal extension is both necessary and sufficient for classic passive chaperone holdase activity. Importantly, we show using thioflavin T binding assays and transmission electron microscopy (TEM) that the IDR of both fly Hsp110 and human Hsp105α and Apg-1 Hsp110 homologs prevents the fibrilization of Aβ_1-42_ and α-synuclein, the highly amyloidogenic peptides involved in Alzheimer’s and Parkinson’s disease pathology, respectively. Our results support the conclusion that metazoan Hsp110 chaperones possess two distinct passive, yet potent, substrate-binding activities and raise the possibility of their exploitation for therapeutic chaperone intervention in age-related neurodegenerative diseases.

## Results

### SBD-β is expendable for Hsp110 chaperoning activity

Previous studies by us and others have established the substrate-binding capacity of the SBD-β subdomain *in vitro* using yeast and mammalian Hsp110 homologs without developing a clear understanding of the biological relevance of this chaperone property (25, 26, 27). To better explore the *in vivo* roles of Hsp110 substrate binding with respect to proteostasis and neurodegenerative disease, we turned our attention to the fruit fly, *D. melanogaster*, which possesses a single Hsp110 homolog encoded by the Hsc70cb gene (30, 31, 32). We purified the Hsc70cb-encoded protein with an added amino-terminal hexa-histidine tag (subsequently termed Hsp110) in *Escherichia coli* cells and utilized a standard spectrophotometric microplate light scattering assay to assess the ability of *Drosophila* Hsp110 to prevent protein aggregation of chemically denatured citrate synthase (CS) (Fig. S1*A*) (33). Hsp110 potently suppressed CS aggregation in a dose-dependent manner with approximately 50% efficacy at a 1:1 ratio of chaperone to substrate. Stronger aggregate inhibition was observed at 2:1 and 4:1 ratios, indicating that Hsp110 prevents aggregation in a manner similar to other homologs (Fig. S1, *B* and *C*) (21, 25, 27, 34). We additionally validated the assay conditions by demonstrating that the nonspecific protein bovine serum albumin (BSA) was unable to prevent CS aggregation (Fig. S2*A*). To verify that the increase in light scatter was indeed indicative of protein aggregation, we subjected samples obtained from the assay after 30 min to differential centrifugation to separate soluble and insoluble materials. While both Hsp110 and BSA remained in the supernatant fraction, only Hsp110 was capable of preventing CS from entering the pellet (Fig. S2*C*).

To further understand the mechanism by which aggregation prevention occurs, a series of deletion mutations were made in the SBD-β subdomain. Five deletion constructs were generated that removed portions or all of SBD-β, including the short hydrophobic linker connecting the NBD to SBD-β (Fig. S3*A*). SBD-β deletion mutants were stably expressed, purified, and screened for chaperone activity (Fig. S3, *B–D*). Surprisingly, we found that partial or complete removal of SBD-β had no significant deleterious effect on aggregation suppression by Hsp110 (Fig. 1, *A*–*C* and Fig. S3, *C* and *D*). Moreover, we noted that removal of SBD-β appeared to significantly enhance Hsp110 chaperoning activity to varying degrees. Interestingly, not only does removal of SBD-β fail to disrupt prevention of CS aggregation, but Hsp110^ΔSBDβ^ appears to be a much more potent holdase than full-length Hsp110 as demonstrated *via* differential centrifugation, with only 4% of CS remaining in the pellet fraction (Fig. 1*D*). To confirm that all our observations were not specific to the CS substrate used in the assays, we repeated the light scatter chaperone experiments using chemically denatured firefly luciferase and obtained similar results, including enhanced chaperone activity at equivalent chaperone to substrate ratios (2:1) (Fig. S4). Together, these data indicate that the *Drosophila* Hsp110 SBD-β is expendable for holdase activity, suggesting the presence of an additional substrate-binding site within the chaperone.

### The C-terminal extension of Hsp110 can operate independently of SBD-β to prevent protein aggregation through an intrinsically disordered region

**Figure 2:**
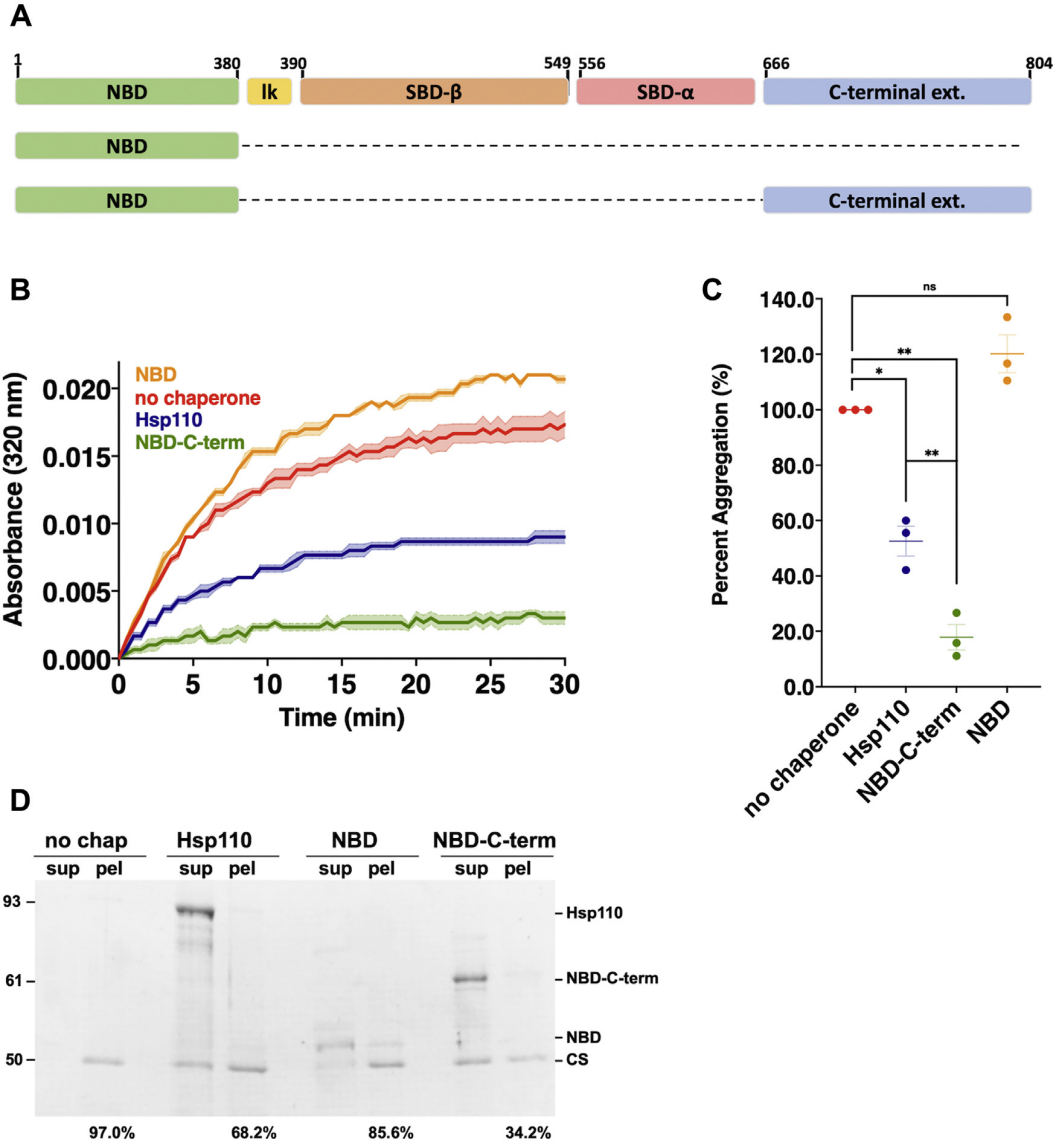
**The far C-terminal extension of *Drosophila* Hsp110 is sufficient for aggregation suppression.***A*, schematic of Hsp110 domain architecture and indicated deletions or fusions. *B*, in total, 200 nM denatured CS was incubated alone (no chaperone) or with 400 nM of respective chaperone: Hsp110, NBD, NBD-C-term. *Bolded lines* are the average of three replicates for each condition while the shaded region represents standard error of the mean (SEM).*C*, end point measurements of each condition were taken from (*A*) and divided by the no chaperone measurement within the respective replicate and converted to relative percentage. Group differences were analyzed using Welch’s *t-*test. ∗*p* = 0.05; ∗∗*p* = 0.005; ∗∗∗*p* = 0.0005; ∗∗∗∗*p* = 0.00005. *D*, following light scatter assay endpoint samples were separated into soluble (sup) and insoluble (pel) fractions by differential centrifugation. Numbers indicate per cent of pellet signal, as quantified using ImageJ, from Coomassie-stained SDS-PAGE gel relative to combined sup plus pel signals.

Previous work using Chinese hamster (*Cricetulus griseus*) Hsp110 demonstrated that the NBD does not possess holdase activity as assessed using firefly luciferase (27). Consistently, we found that purified *Drosophila* Hsp110 NBD likewise was incapable of preventing aggregation of CS as demonstrated using both light scattering and differential centrifugation assays (Fig. 2). We noted that unlike the yeast Hsp110 Sse1, *Drosophila* Hsp110 possesses a carboxyl-terminal extension (C-term) of approximately 106 additional amino acids (Fig. S5). To assess whether this previously unstudied region may contribute to chaperone activity, we fused the C-term to the NBD and tested this purified protein for holdase function (Fig. S1*A*). Remarkably, the NBD–C-term hybrid protein exhibited potent aggregate suppression activity (Fig. 2, *A*–*C*). Differential centrifugation confirmed the light scatter assay results, demonstrating that all of the NBD–C-term protein and the majority of unfolded CS remained in the soluble fraction after a 30 min incubation (Fig. 2*D*). Once again, we noted that like Hsp110^ΔSBDβ^, NBD-C-term appeared to be more efficient at aggregation prevention than full-length Hsp110. These findings demonstrate for the first time a second, previously unknown chaperoning site within a metazoan Hsp110 homolog that is capable of functioning independently of the canonical β-sandwich region (21, 27).

**Figure 3:**
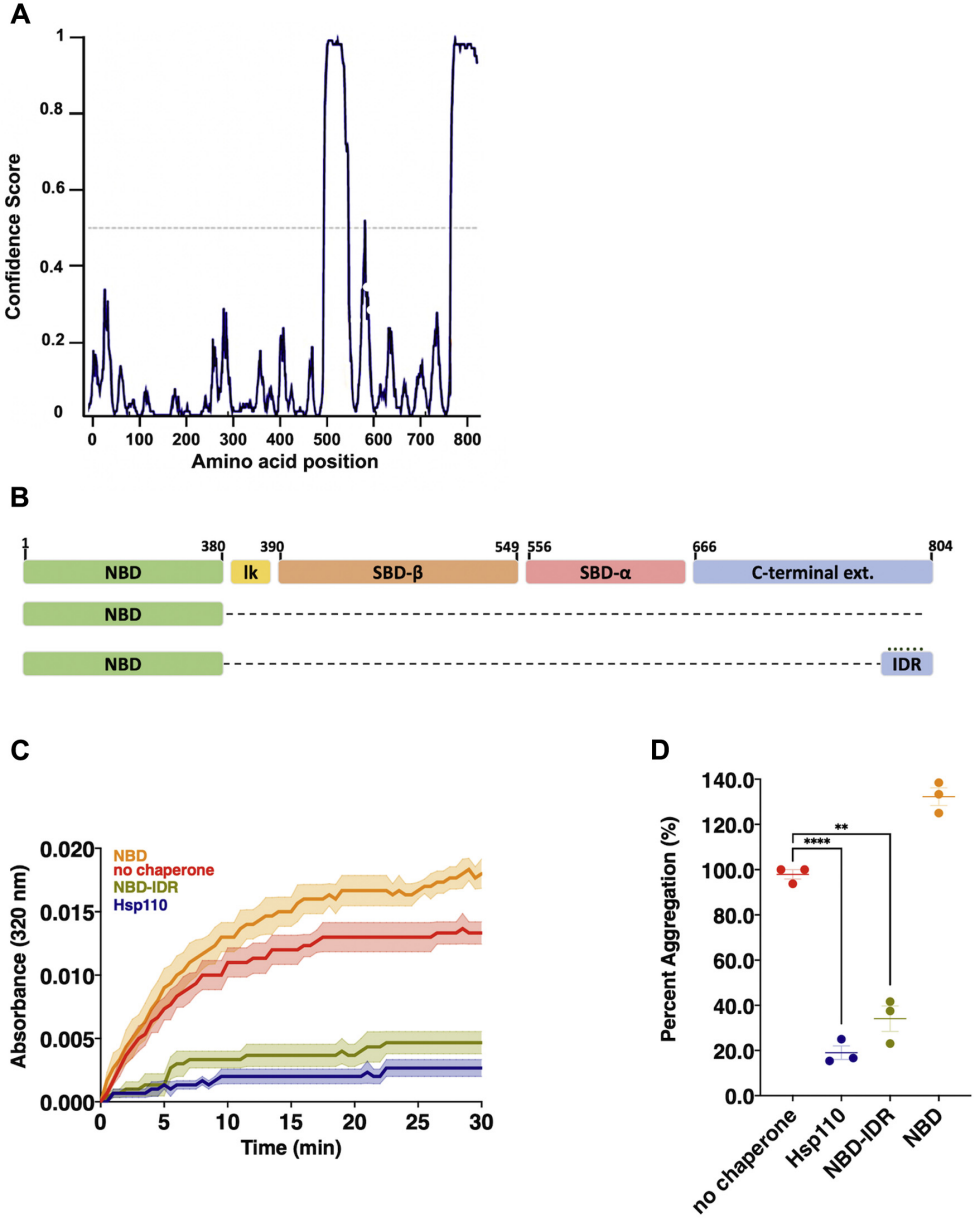
**A predicted intrinsically disordered region (IDR) within the far C-terminal extension mediates aggregation suppression.***A*, disorder prediction of *Drosophila* Hsp110 using DISOPRED3. Peaks above the dotted line (>0.5 confidence score) indicate regions of predicted disorder. *B*, schematic of Hsp110 domain architecture and indicated deletions or fusions. *C*, in total, 200 nM denatured CS was incubated alone (no chaperone) or with 400 nM of respective chaperone: Hsp110, NBD, NBD-IDR. *Bolded lines* are the average of three replicates for each condition while the shaded region represents standard error of the mean (SEM). *C*, end point measurements of each condition were taken from (*B*) and divided by the no chaperone measurement within the respective replicate and converted to relative percentage. Group differences were analyzed using Welch’s *t-*test. ∗*p* = 0.05; ∗∗*p* = 0.005; ∗∗∗*p* = 0.0005; ∗∗∗∗*p* = 0.00005.

To more precisely map sequences conferring holdase activity within the C-term, we explored the *Drosophila* Hsp110 protein sequence to identify potential structural element boundaries to aid in design of additional deletions and truncations. As no full-length structures are available for *Drosophila* or any other metazoan Hsp110 homologs, we turned to *in silico* analysis. Using the protein disorder prediction package DISOPRED3, we identified two potential regions with high confidence scores for intrinsic disorder and the potential for protein binding (Fig. 3*A*) (35, 36). Of the two sites identified by the prediction software, one mapped to the extended loop region (residues 508–544) within SBD-β that differentiates this domain from the more distantly related Hsp70 superfamily homologs and another to the terminal 53 amino acids (residues 751–804) of the protein localized within the C-term region demonstrated to possess chaperone activity in Figure 2 (Fig. 3*B*). Previous studies have revealed that IDRs can act as sites of protein binding, assuming order when interacting with other proteins and peptides (37). This property is exploited by some protein chaperones. For example, the antiaggregation/sequestration activity of the small heat shock protein (sHsp) family is mediated in part by IDRs (38, 39, 40, 41). The localization of this IDR within the C-terminal extension made it a region of interest as a potential site for substrate–chaperone interaction. We therefore appended the predicted IDR to the NBD to create NBD-IDR and found that the IDR conferred robust aggregation prevention to the NBD (Fig. 3, *C* and *D*). The holdase activity observed in the C-term domain of Hsp110 is thus due to the 53 amino acids composing a predicted IDR at the extreme carboxyl terminus. Overall, these results confirm the presence of substrate-binding capacity in the C-terminal extension mediated by a 53-amino-acid IDR.

### The novel IDR-containing C-terminal extension is conserved in metazoan Hsp110s, Apg-1 and Hsp105α

**Figure 4:**
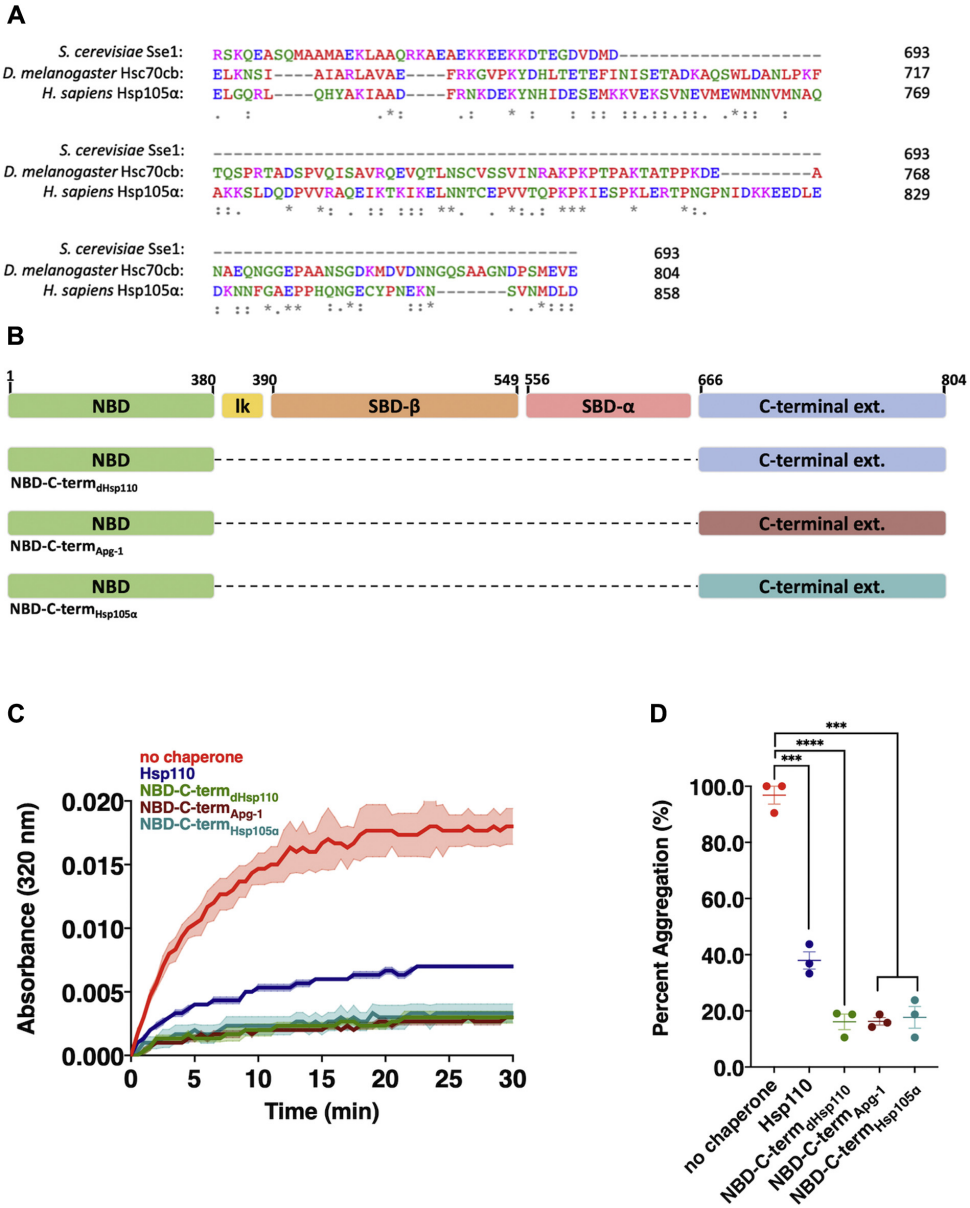
**Aggregation suppression by conserved C-terminal extensions in human Hsp110 homologs Apg-1 and Hsp105α.***A*, clustal Omega sequence alignment of the carboxyl termini of Sse1 (budding yeast), Hsc70 cB (fruit fly), and Hsp105α (human) Hsp110 homologs. *B*, schematic of Hsp110 domain architecture and indicated deletions or fusions. *C*, in total, 200 nM denatured CS was incubated alone (no chaperone) or with 400 nM of respective chaperone: Hsp110, NBD-C-term_dHsp110_, NBD-C-term_Apg-1_, NBD-C-term_Hsp105α_. *Bolded lines* are the average of three replicates for each condition while the shaded region represents standard error of the mean (SEM). *D*, end point measurements of each condition were taken from (*B*) and divided by the no chaperone measurement within the respective replicate and converted to relative percentage. Group differences were analyzed using Welch’s *t-*test. ∗*p* = 0.05; ∗∗*p* = 0.005; ∗∗∗*p* = 0.0005; ∗∗∗∗*p* = 0.00005.

Clustal Omega sequence alignment revealed that the C-terminal extension is present in several metazoan Hsp110 homologs including bovine, chimpanzee, orangutan, and the African clawed frog, among others (Fig. S5) (42). Notably, the C-term extension and IDR appear to be conserved in the human Hsp110 homologs Apg-1and Hsp105α (Figs. S5 and 4*A*). While these regions are not strictly conserved among species in terms of sequence, there appears to be conservation in terms of amino acid characteristics and biochemical properties (43). Previous *in vitro* studies have demonstrated that both murine Hsp105α and Apg-1 possess passive chaperone holdases activity as we have discovered in the *Drosophila* Hsp110 (27, 44). We therefore constructed and purified chimeric versions of the NBD-C-term constructs in which the *Drosophila* Hsp110 NBD was fused to the C-terminal extensions of either human Hsp105α or Apg-1 (Fig. S1*A*). Using the light scatter CS aggregation assay, we found that both Apg-1 and Hsp105α C-terminal extensions, when fused to the *Drosophila* NBD, robustly prevented protein aggregation (Fig. 4, *B*–*D*). Additionally, Apg-1 and Hsp105α C-terminal extensions appeared to be slightly more efficient holdases than full-length fly Hsp110. These results confirm that the human Hsp110s contain conserved C-terminal extensions that act as potent passive chaperone regions, presumably through the IDR domains, and suggest potential evolutionary advantage of this region in animals.

### Human and *Drosophila* Hsp110 IDRs prevent amyloid formation

**Figure 5:**
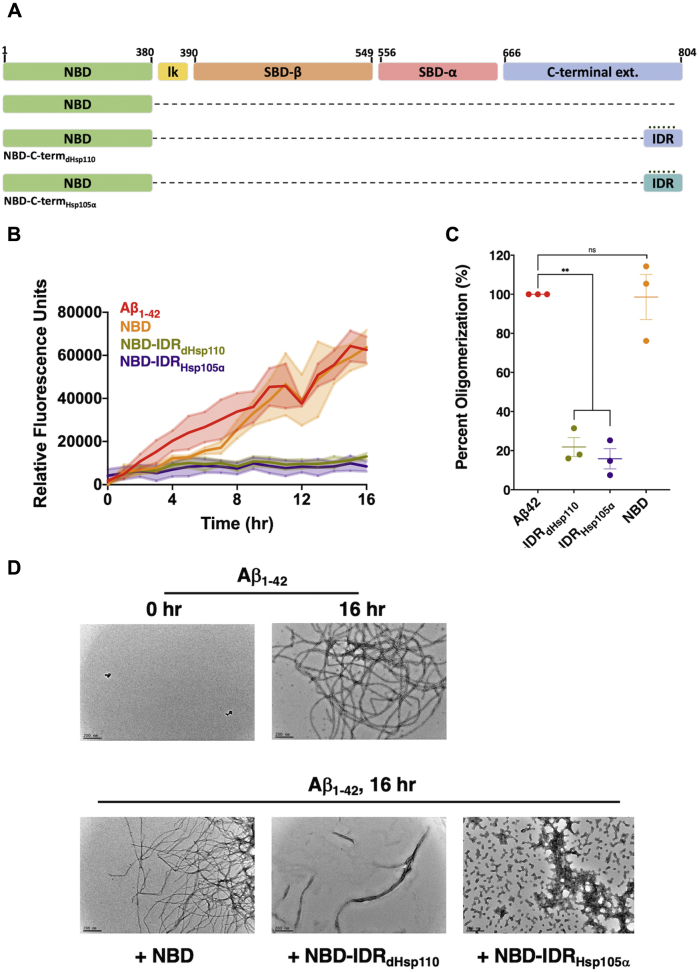
***Drosophila* and human IDRs prevent oligomerization and fibril formation of Alzheimer’s peptide amyloid β.***A*, schematic of Hsp110 domain architecture and indicated deletions or fusions. *B*, in total, 2 μM of Aβ42 was incubated alone (Aβ42 only) or with 4 μM of respective chaperone: NBD, NBD-IDR_dHsp110_, NBD-IDR_Hsp105α_ in the presence of the amyloid-detecting dye thioflavin T for the indicated time course and fluorescence detected as described in Experimental procedures. *Bolded lines* are the average of three replicates for each condition while the shaded region represents standard error of the mean (SEM). *C*, End point measurements of each condition were taken from (*A*) and divided by the no chaperone measurement within the respective replicate and converted to relative percentage. Group differences were analyzed using Welch’s *t-*test. ∗*p* = 0.05; ∗∗*p* = 0.005; ∗∗∗*p* = 0.0005; ∗∗∗∗*p* = 0.00005. *D*, end point samples from the thioflavin T binding assay were recovered, negative stained, and imaged *via* transmission electron microscopy, with or without additional chaperones as indicated. *Scale bar* = 200 nm.

The presence of the C-terminal extension and the conservation of this substrate-binding capacity in human Hsp110s prompted us to ask what potential it has for acting on proteins implicated in human neurodegenerative disease linked to protein conformation (13, 16, 17, 18, 45). Amyloid β (Aβ) is a cleavage product of the amyloid precursor protein (APP) and a major component of amyloid plaques associated with Alzheimer’s disease toxicity (46, 47, 48). APP cleavage creates two forms of amyloid β peptide, Aβ_1-42_ and Aβ_1-40_, of which Aβ_1-42_ is the more toxic and amyloidogenic form (49). Aβ peptides will assemble over time into oligomeric species that ultimately form large fibrils that comprise the amyloid plaques (50, 51). The fibrilization reaction can be followed over time *in vitro* by assessing binding of the fluorescent dye thioflavin T, which selectively binds β-sheets in amyloid structures (52, 53). We therefore developed a microplate thioflavin T-based fibrilization assay and observed significant signal production from commercially obtained Aβ_1-42_ monomers over a 16 h time course (Fig. 5, *A*–*C*). Addition of the NBD had little impact on fibrilization yield or kinetics. We generated two new chimeric protein fusions in which only the predicted IDRs of both *Drosophila* Hsp110 and human Hsp105α were fused to the *Drosophila* NBD and found that in contrast to NBD alone, both the IDR fusions completely prevented the fibrilization of Aβ_1-42_. Consistent with previous reports, the addition of an Hsp110 chaperone, in this case the minimal chaperoning entity NBD-IDR_Hsp105α_, in the absence of Hsp70 and Hsp40 did not resolubilize fibrils that have already formed (Fig. S6) (14, 44).

Aggregation and plaque formation of the protein α-synuclein (α-syn) are a major contributor to Parkinson’s disease. Fibrilization of α-syn can also be tracked *via* thioflavin T fluorescence but typically requires seeding by preformed fibrils (PFF) in *in vitro* assay systems. We obtained commercially prepared α-syn monomers and PFF and observed modest thioflavin T fluorescence over a 24 h time course from the PFF but not the monomers (Fig. S7). Addition of PFF to α-syn monomers resulted in a slow increase in thioflavin T fluorescence over 24 h in a manner that was accelerated by the further addition of the *Drosophila* NBD by an unknown mechanism with no difference in overall signal yield. Remarkably, both NBD-IDR fusions allowed a small increase in fluorescence over the first hour of the experiment but little further increase, demonstrating highly effective suppression of α-syn fibrilization (Fig. S7, *B* and *C*).

To further assess the ability of C-terminal IDRs to prevent fibrilization transmission, we turned to TEM, which allows visualization of amyloid Aβ_1-42_ structures. Samples were removed from the microplate wells after the 16 h thioflavin T experiment and deposited onto grids for TEM. Extensive fibrilization of Aβ_1-42_ was observed after 16 h that was absent at the initiation of the assay (Fig. 5*D*, *top panels*). Incubation of Aβ_1-42_ with both NBD-IDRs drastically decreased formation of higher order Aβ_1-42_ fibrils while addition of the NBD alone had no impact on fibril formation (Fig. 5*D*, *bottom panels*). The fibrils that did form in the presence of the IDRs were predominantly shorter and smaller than the fibrils seen with Aβ_1-42_ at 16 h. We noted that in the presence of NBD-IDR_Hsp105α_, but not NBD-IDR_dHsp110_, numerous small globular structures were observed. To further investigate this phenomenon, we incubated NBD-IDR_Hsp105α_ alone for 16 h under the same conditions and observed the same particles that averaged approximately 30 nm in diameter, indicating that the chaperone fusion alone is capable of forming these assemblies (Fig. S8). Together these experiments establish that the C-terminal IDRs of *Drosophila* and human Hsp110 homologs are capable of inhibiting fibrilization of two critical amyloidogenic substrates *in vitro*, strongly suggesting that this domain may impart similar activity to the Hsp110 in flies and humans.

## Discussion

In this study we reveal a novel aggregation prevention mechanism in metazoans provided by a previously unstudied region at the far C-terminus of the Hsp110 family of molecular chaperones. This antiaggregation property is mediated by predicted IDRs within the C-terminal extension and includes amino acids 718–804 in *Drosophila* Hsc70cb and 803–858 in human Hsp105α. Additionally, IDR-mediated chaperoning can prevent amyloid formation of Aβ_1-42_ and α-synuclein, the causative agents of Alzheimer’s and Parkinson’s diseases, respectively. These functions do not require the presence of the canonical SBD-β, establishing the presence of a second chaperone site within this member of the Hsp70 chaperone superfamily.

IDRs are protein segments that do not maintain a fixed structure in their native states (54). IDRs tend to be low in amino acid complexity and sequence conservation. Nonetheless, their abundance in eukaryotic species indicates that they serve an essential purpose (55). IDRs have been shown to assume secondary and tertiary structures when interacting with other polypeptides (56). The abundance of IDRs in nature, particularly in higher-order organisms, has prompted investigation into the functional relevance of these sites in protein–protein interactions (57). Previous work supports the function of sHsps being due in part to IDRs facilitating chaperoning activities (37, 58). sHsps including yeast Hsp42 act as sequestrases, corralling misfolded proteins to prevent their aggregation until the Hsp70 machinery can bind and facilitate proper folding conformations or target substrates to degradation pathways (59). In a similar manner, the Hsp110 NBD-IDR constructs appear to function by binding to unfolded and amyloidogenic proteins in an ATP-independent manner, preventing aggregation and maintaining these proteins in a soluble state. It is possible that IDR in full-length Hsp110 provides a similar function in addition to the well-established nucleotide exchange activity characteristic of this family of chaperones. Amino acid composition analysis as well as homology searches revealed no consistent or conserved patterns in the fly or human IDR sequences that might provide mechanistic insight, beyond enrichment in glycine, proline, and negatively charged residues. Indeed, the sequence conservation between the *Drosophila* and higher metazoan IDRs is quite low. Further biochemical and biophysical analysis of this domain will be required to understand its mode of operation.

While the resolution of the light scatter assays is somewhat limited, it appears that chimeric proteins consisting of either the entire C-terminal extension or just the IDR appended to nonchaperoning NBD prevent aggregation in a capacity similar to, and in some cases better than, full-length Hsp110. It is unclear why this is the case, but we speculate it may be due to differential accessibility of the IDRs. It is possible that the folding of full-length Hsp110 is such that it partially obscures the C-terminal extension, making it harder to fully interact with misfolded polypeptides. Interestingly, removal of some or all of SBD-β also enhanced inhibition of CS aggregation in the context of the otherwise full-length Hsp110 protein (Fig. 1*B* and Fig. S3, *C* and *D*). Deletion of this domain may cause changes in protein structure such that the IDR is fully exposed and more available to interact with substrates. Alternatively, SBD-β itself may interact with the IDR or otherwise occlude the IDR from productive substrate interaction. An interesting ancillary observation is the assembly of NBD-IDR_Hsp105α_ into what appear to be globular particles. Initially we speculated that these images represented NBD-IDR_Hsp105α_ oligomerizing in concert with Aβ_1-42_ oligomers or protofibrils. However, NBD-IDR_Hsp105α_ clearly assembled into the same particles in the absence of Aβ_1-42_ (Fig. S8). Additionally, Aβ_1-42_ is typically found in fibril rather than spherical oligomeric states, making it unlikely that the substrate is dictating the NBD-IDR ultrastructure. TEM images of the classic sHsp αB-crystallin highly resemble the NBD-DR particles, with similar structure and diameter (60). It is therefore possible that these particles represent an alternative native conformation of NBD-IDR_Hsp105α_ that is not a prerequisite for chaperone activity as we did not observe the same behavior for NBD-IDR_dHsp110_. As there are no reports of native human Hsp110 chaperones assembling into such particles, it is also likely that they are a by-product of an “unrestricted” IDR.

We have demonstrated that the C-terminal IDRs are potent suppressors of Aβ_1-42_ and α-synuclein oligomerization *in vitro*. Overexpression of Hsp110 in several different biological systems has been reported to reduce formation of toxic misfolded protein conformers (13, 15, 16, 17, 18). These findings are at odds with the known inhibitory effect that Hsp110 has on Hsp70 ATPase activity and thus protein folding when present above a roughly 1:10 ratio (110:70) due to excessive NEF activity. Moreover, our *in vitro* experiments firmly establish that the Hsp110 C-terminal IDR is capable of suppressing aggregate and amyloid formation in the absence of Hsp40 and Hsp70. Thus, while modulation of Hsp70 substrate cycling may be one mechanism by which Hsp110 contributes to protein homeostasis, another Hsp70-independent mechanism clearly exists. We are currently attempting to build *in vivo* evidence for roles for both SBD-β and the C-terminal IDR in metazoan neurogenerative disease onset and progression using *Drosophila* amyloidogenesis models.

It is tempting to speculate that our findings suggest a potential functional therapeutic for human neurodegenerative disease. Engineered chaperone overexpression to combat diseases of protein misfolding is fraught with nonspecific and collateral effects due to the key roles chaperones play in all cellular biology (61, 62, 63). It is possible that further investigations into using the Hsp110 IDR peptide alone or attached to an inert protein scaffold may yield a deliverable tool that would block or delay fibril and plaque formation in several amyloidogenic disorders.

## Experimental procedures

### Plasmid construction

Hsc70cb cDNA was amplified using PCR and inserted into the pProEX-HTA protein expression vector using 5’ SacI and 3’ SpeI restriction enzyme sites (Invitrogen). Two *Drosophila* NBD constructs (with or without a stop codon) were created by PCR amplification followed by cloning into pProEX-HTA using 5’ SacI and 3’ SpeI restriction sites. The *Drosophila* NBD–C-term construct was designed and ordered from Genewiz and subcloned into pProEX-HTA using 5’ SacI and 3’ SpeI restriction sites. Apg-1 and Hsp105α NBD-C-term constructs were created by PCR amplification of the respective C-terminal extensions from cDNA and insertion into the nonstop *Drosophila* pProEX-HTA-NBD plasmid using 5’ Spe1 and 3’ XhoI restriction sites. NBD-IDR constructs were created by PCR amplification of Hsc70cb and Hsp105α C-terminal IDRs, followed by cloning into the nonstop *Drosophila* pProEX-HTA-NBD plasmid using 5’ SpeI and 3’ XhoI restriction sites.

### Protein purification

BL21 *E. coli* cells were used to express and purify all chaperones and fusion proteins used in this study. Subculture of overnight inoculum was made to an OD of 0.15 in 600 ml Luria Broth with ampicillin (100 μg/ml) and grown to OD 0.6. Isopropyl β-d-1-thiogalactopyranoside was added to the culture flask to a final concentration of 1 mM and flasks were subject to shaking with aeration for 4 h at 25 °C. Cell pellets were collected and flash frozen until processing for immobilized affinity chromatography (iMAC). For iMAC, cells were thawed and lysed using chemical lysis Buffer B (50 mM Tris Base - pH 7.5, 5 mM imidazole, 2 mM MgCl_2_, 200 mM NaCl, 10% octylthioglucoside). Lysates were incubated with Talon cobalt resin (Takara Bio) for 1 h at 4 °C with top-over-bottom mixing. Following incubation, resin was separated from lysates using centrifugation, washed with Buffer B and Buffer C (50 mM Tris - pH 7.5, 10 mM imidazole, 2 mM MgCl_2_, 600 mM NaCl), and chaperones eluted with Buffer E (50 mM Tris - pH 7.5, 200 mM imidazole, 2 mM MgCl_2_, 700 mM NaCl). Eluates were concentrated to ∼500 μl and kept at –80 °C until further purification *via* size-exclusion chromatography (SEC). iMAC samples were thawed on ice and loaded into a purification column packed with 10 ml of Sephacryl S-100 (GE Healthcare) equilibrated with 25 mM Tris-HCl, pH 7.5100 mM NaCl buffer. Samples were eluted in the same buffer and peak fractions collected and frozen at –80 °C until use.

### Light scatter aggregation assay

Stock CS (Sigma-Aldrich) and firefly luciferase solutions were diluted to 18.7 μM using a chemical denaturing buffer (6.6 M guanidine hydrochloride, 5.5 mM dithiothreitol) and incubated for 1 h at 25 °C before assays. Aggregation assays were performed by incubating 200 nM denatured CS or firefly luciferase with 400 nM of respective chaperones, chimeric proteins, or bovine serum albumin in a refolding buffer (25 mM Tris-HCl, pH 7.5, 100 mM NaCl) in a total volume of 180 μl in a clear-bottom 96-well plate. An MX Synergy (BioTek) plate reader was used to obtain 320 nm absorbance readings every 30 s for 30 min (CS), or 90 min for firefly luciferase, at 25 °C. Assays were performed in technical triplicate for each experiment and averaged for three separate experimental replicates.

### Differential centrifugation

To quantify the amount of soluble *versus* insoluble protein after the aggregation assays, 175 μl of the reaction was taken at the conclusion of the time course. This fraction was subjected to differential centrifugation at 16,000*g* for 4 min. The supernatant (169 μl) was removed to a new tube and the remaining pellet fraction was brought to 169 μl with refolding buffer. Both supernatant and pellet fractions were brought to 400 μl volume with molecular biology-grade water and 40 μl of trichloroacetic acid was added to precipitate proteins. After a 45 min incubation samples were spun at 16,000*g* for 15 min, washed with acetone, dried, and resolubilized in 15 μl SDS-PAGE sample buffer and fractions analyzed using SDS-PAGE and Coomassie staining. Amount of substrate in each fraction as a percent of total was quantified using ImageJ software (NIH).

### Thioflavin T binding assay

α-Synuclein thioflavin T binding assays were performed by incubating 2 μM α-synuclein monomer (StressMarq Biosciences), 1 μM preformed fibrils (StressMarq Biosciences), 4 μM of each respective chaperone, and 5 μM thioflavin T (Sigma) in a total reaction volume of 180 μl using a 96-well μClear bottom black plate (Greiner Bio-One). Samples were subject to excitation at 450 nm and emission at 490 nm at 37 °C and fluorescence intensity was measured every 5 min for 24 h using an MX Synergy microplate reader. Aβ_1-42_ was purchased from GenScript USA, solubilized from lyophilized powder into 0.1 M NaOH, pH 12.0, and frozen in aliquots at –80 °C to maintain the peptide as a monomer. Thioflavin T binding assays were performed by incubating diluted 2 μM Aβ_1-42_, 4 μM of each respective chaperone, and 5 μM thioflavin T in a total reaction volume of 50 μl containing 100 mM NaCl and 25 mM Tris-HCl, pH 7.5, in a 96-well μClear bottom black plate. Fluorescence intensity measurements (ex. 450/em. 490) were taken every 5 min for 16 h at 37 °C using an MX Synergy microplate reader.

### Disaggregation assay

Aβ_1-42_ disaggregation assay was performed by incubating 2 μM Aβ_1-42_ and 5 μM thioflavin T in 100 mM NaCl and 25 mM Tris-HCl, pH 7.5 in a total reaction volume of 50 μl in a 96-well μClear bottom plate. Fluorescence intensity measurements (ex. 450/em. 490) were taken every 5 min for 20 h at 37 °C using a BioTek MX Synergy microplate reader. At 20 h, 4 μM of NBD-IDR_Hsp105α_ was added and the reaction continued for an additional 6 h.

### Transmission electron microscopy

Samples were prepared by taking baseline fluorescence measurements of all conditions and removing Aβ_1-42_ at T = 0. The remaining samples were measured by the protocol detailed in the thioflavin T binding assay section after which samples were recovered and diluted to a final protein concentration of 1.0 μM. Samples were negatively stained with 1% uranyl acetate in Tris NaCl refolding buffer and fixed onto 400 mesh copper grids (Electron Microscopy Sciences, Hatfield, PA). TEM imaging was done at 100× magnification with a JEOL 1400 electron microscope. Representative images were used for all conditions. Measurement of particles observed with the NBD-IDR_Hsp105α_ chimeric protein was performed using the distance tool on pixel-calibrated images using ImageJ.

### Statistical analysis

Welch’s variance *t-*test of unequal variance was used to analyze the mean differences between conditions. End point measurements for each replicate in a condition were averaged and analyzed using Prism 9 (Graphpad Software). For all time course experiments measurements at each time point were taken and averaged and the standard error of the mean was calculated. For the plots darker solid lines represent the calculated mean while the light shading represents the standard error of mean for those data. For all significance tests, ∗*p* = 0.05; ∗∗*p* = 0.005; ∗∗∗*p* = 0.0005; ∗∗∗∗*p* = 0.00005.

## Data availability

All relevant data are contained within the article and supporting information.

## Supporting information

This article contains supporting information.

## Conflict of interest

The authors declare that they have no conflicts of interest with the contents of this article.
