# Supplementary material for: Suppression of aggregate and amyloid formation by a novel intrinsically disordered region in metazoan Hsp110 chaperones
Source: J Biol Chem. 2021 Mar 19;296:100567. doi: 10.1016/j.jbc.2021.100567 (PMC8063735; doi:10.1016/j.jbc.2021.100567)
Supplement: Figures S1 to S8 [file mmc1.pdf]

SUPPLEMENTAL INFORMATION

"Suppression of aggregate and amyloid formation by a novel intrinsically disordered region in metazoan Hsp110 chaperones "

Yakubu and Morano, 2021

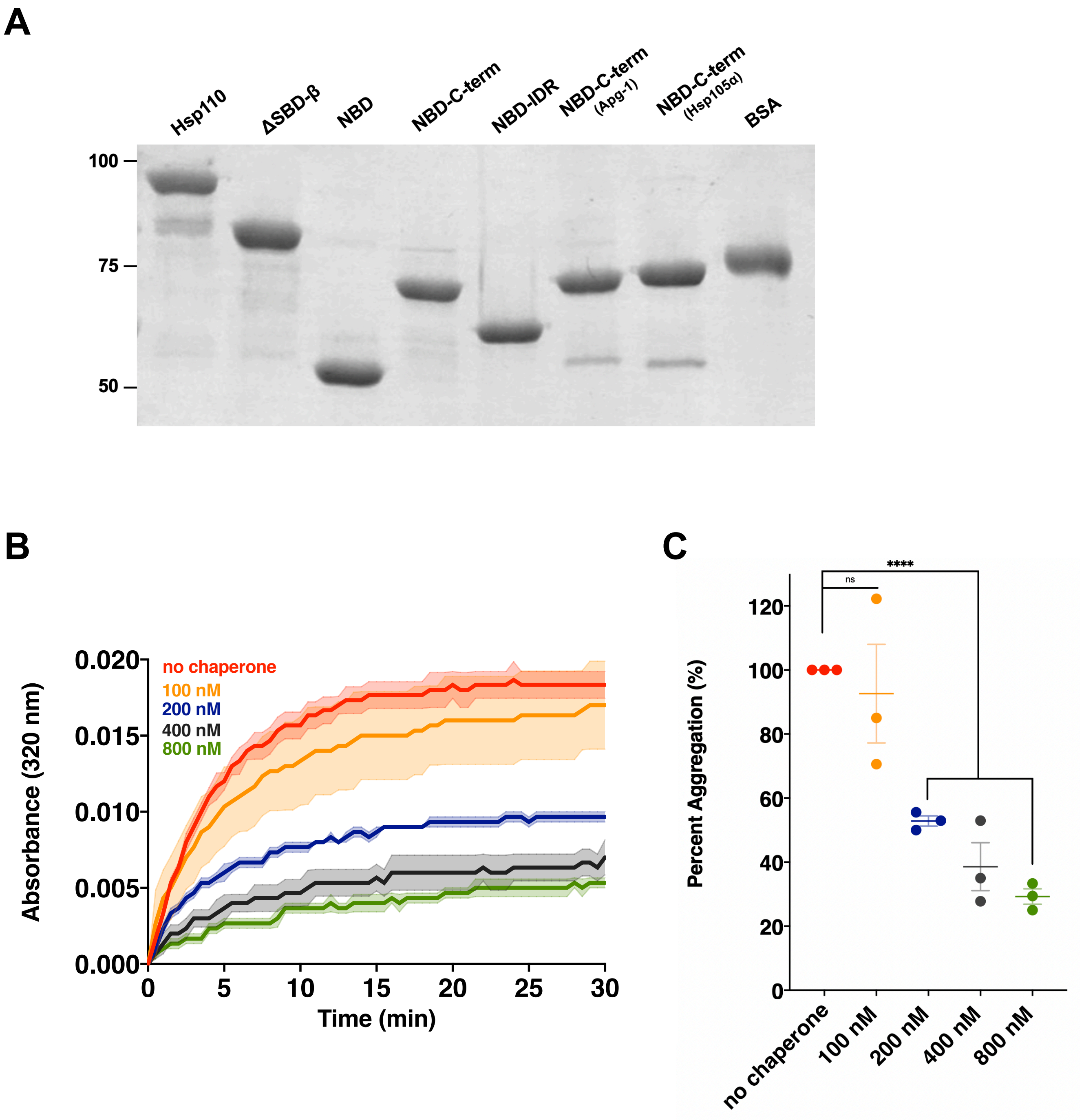

Figure S1: Hsp110 prevents aggregation in a dose-dependent manner. **A.** 500 ng of each purified chaperone variant used in this study shown via 12% SDS-PAGE stained with Coomassie Brilliant Blue. **B.** 200 nM denatured CS was incubated alone (no chaperone) or with *Drosophila* Hsp110 at **.5x**, **1x**, **2x**, or **4x** concentration. Bolded lines are the average of three replicates for each condition while the shaded region represents standard error of the mean (SEM). End point measurements of each condition were taken from (Fig. S1B) and divided by the no chaperone measurement within the respective replicate and converted to relative percentage. Group differences were analyzed using Welch's *t*-test. *, p=0.05; **, p=0.005; ***, p=0.0005; ****, p=0.00005.

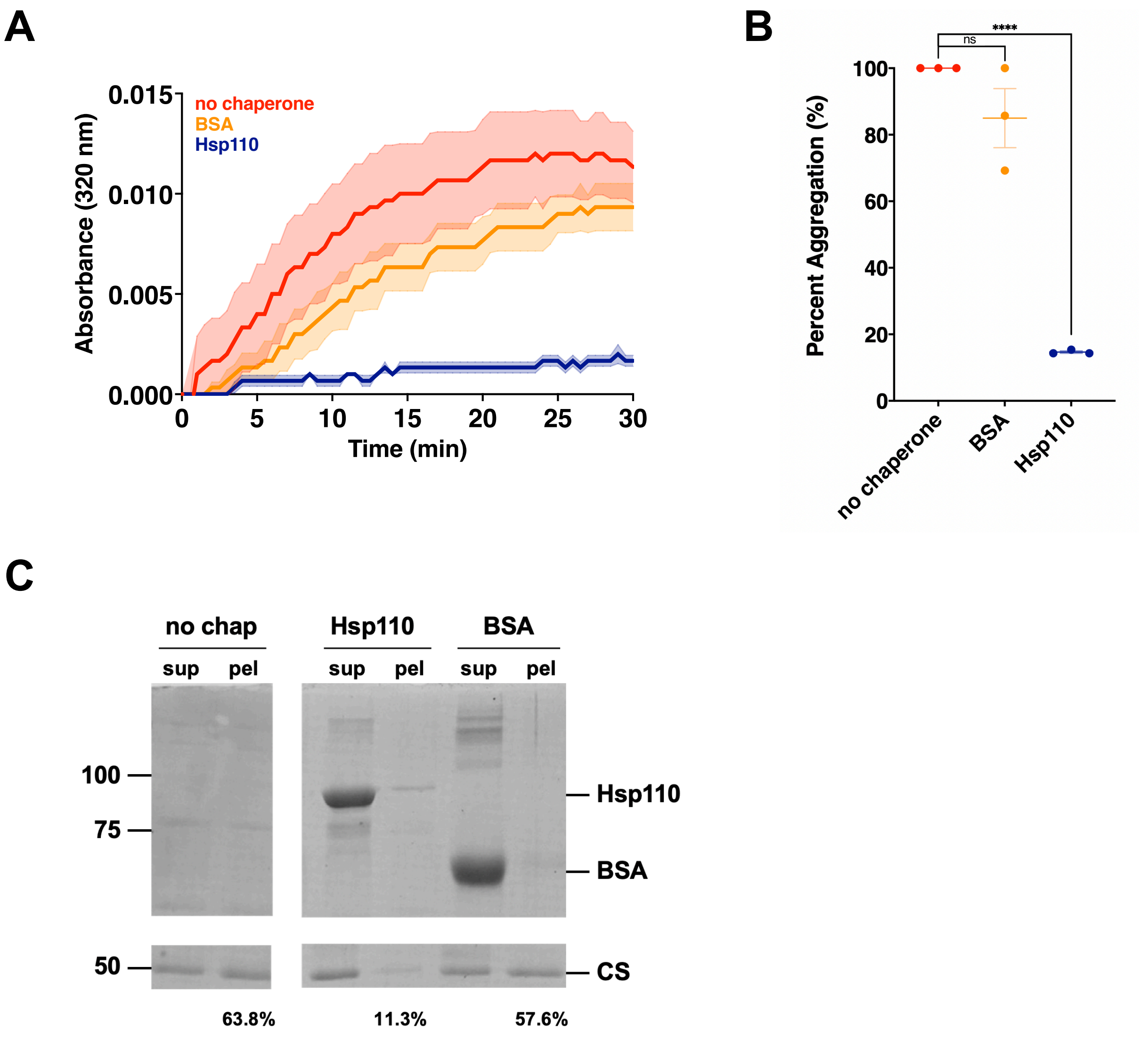

Figure S2: Non-chaperone protein bovine serum albumin (BSA) does not prevent CS aggregation. A. 200 nM denatured CS was incubated alone (no chaperone) or with 400 nM Hsp110 or BSA. **B.** End point measurements of each condition were taken from (Fig. S2A) and divided by the no chaperone measurement within the respective replicate and converted to relative percentage. Group differences were analyzed using Welch's *t*-test. *, p=0.05; **, p=0.005; ***, p=0.0005; ****, p=0.00005. **C.** Following light scatter assay endpoint samples were separated into soluble (sup) and insoluble (pel) fractions by differential centrifugation. Numbers indicate per cent of pellet signal, as quantified using ImageJ, from Coomassie-stained SDS-PAGE gel relative to combined sup plus pel signals.

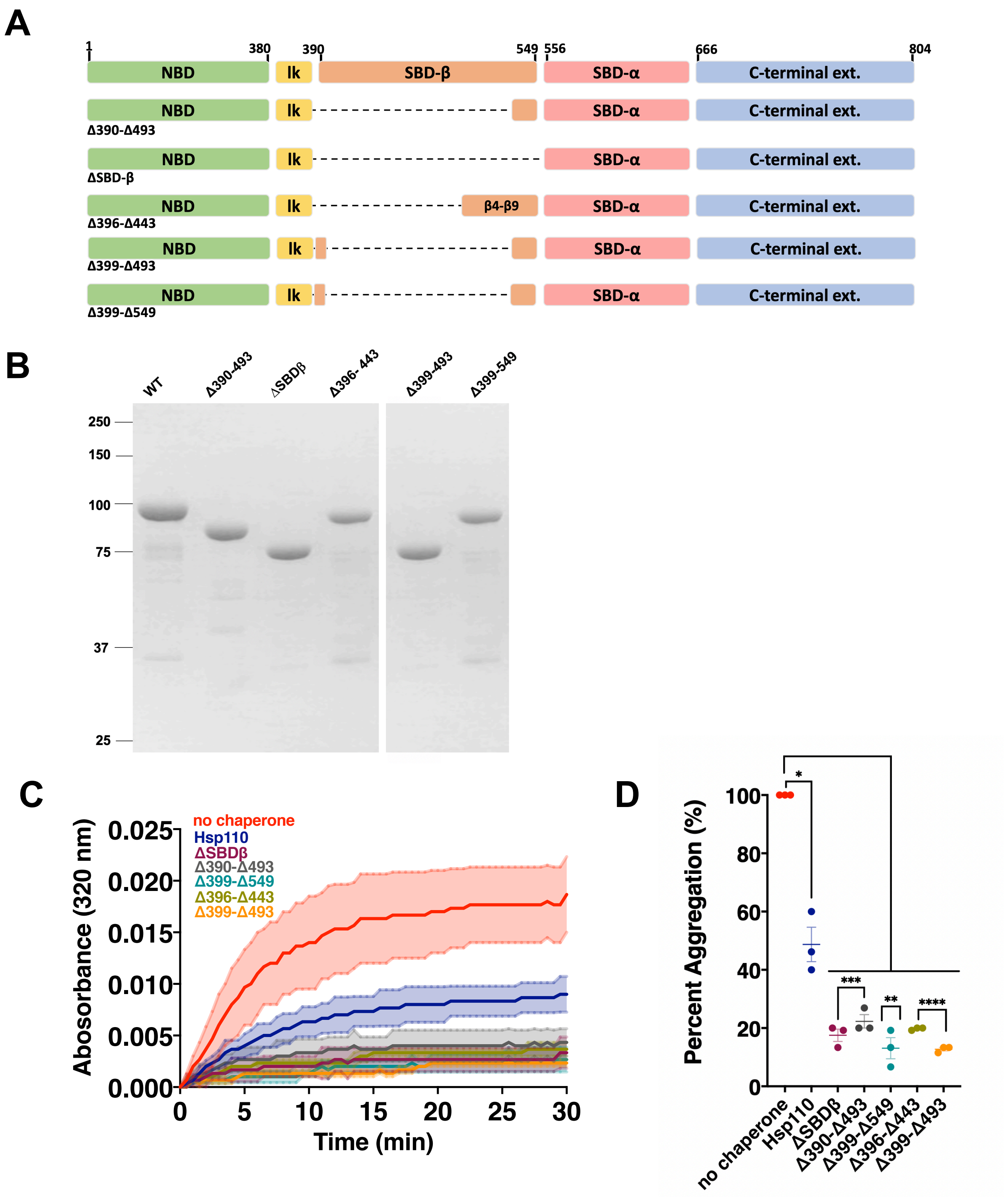

Figure S3: Partial or complete deletion of SBD-β does not eliminate Hsp110 substrate binding activity. A. Schematic of Hsp110 domain architecture indicating various SBD-β deletion mutants. **B.** 200 ng of purified Hsp110 and each SBD-β deletion mutant used in this study via 12% SDS-PAGE stained with Coomassie Brilliant Blue. **C.** 200 nM denatured CS was incubated alone (no chaperone) or with 400 nM of respective chaperone: Hsp110, Hsp110$^{\Delta SBD\beta}$, Hsp110$^{\Delta 390\text{-}493}$, Hsp110$^{\Delta 399\text{-}549}$, Hsp110$^{\Delta 396\text{-}443}$, Hsp110$^{\Delta 399\text{-}493}$. Bolded lines are the average of three replicates for each condition while the shaded region represents standard error of the mean (SEM). **D.** End point measurements of each condition were taken from (Fig. S3C) and divided by the no chaperone measurement within the respective replicate and converted to relative percentage. Group differences were analyzed using Welch's *t*-test. *, p=0.05; **, p=0.005; ***, p=0.0005; ****, p=0.00005.

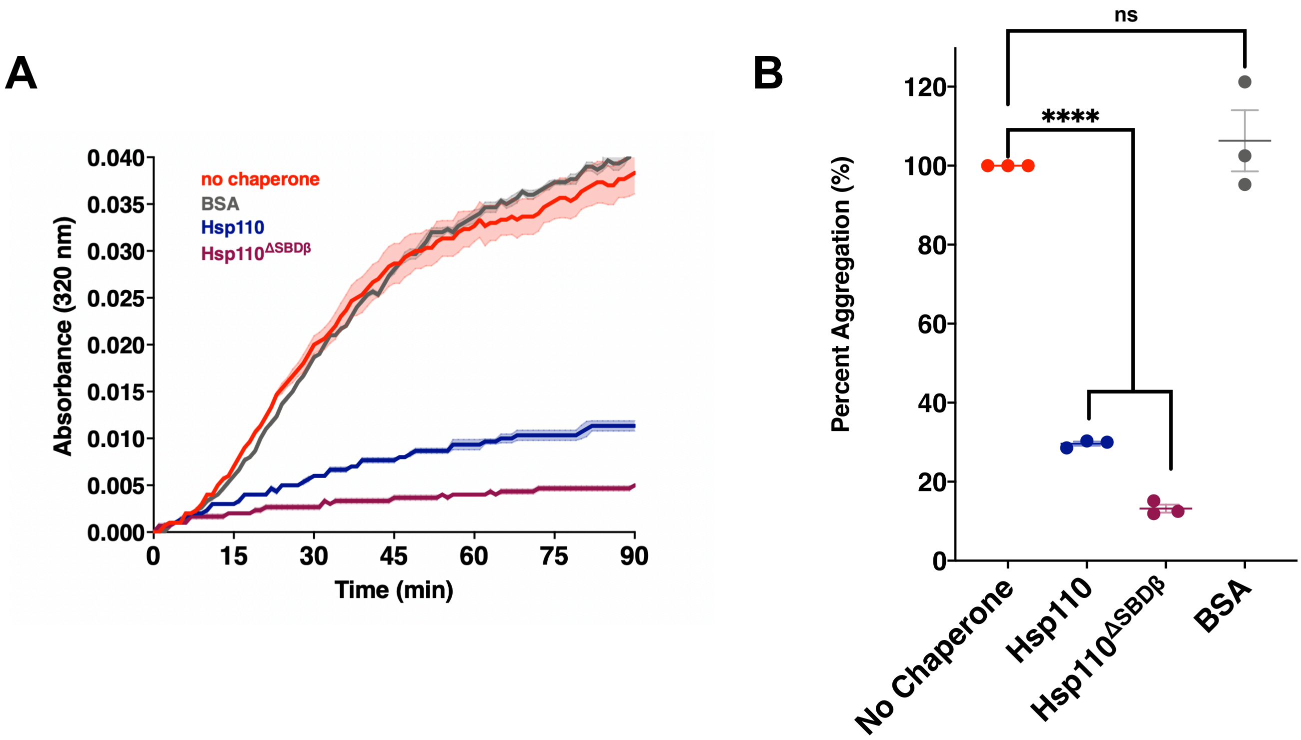

Figure S4: SBDb is not required for aggregation prevention of denatured firefly luciferase. A. 200 nM denatured firefly luciferase was incubated alone (no chaperone) or with 400 nM of respective chaperone/protein: Hsp110, $Hsp110^{\Delta SBD\beta}$, BSA**.** Bolded lines are the average of three replicates for each condition while the shaded region represents standard error of the mean (SEM). **B.** End point measurements of each condition were taken from (Fig. S4A) and divided by the no chaperone measurement within the respective replicate and converted to relative percentage. Group differences were analyzed using Welch's *t*-test. *, p=0.05; **, p=0.005; ***, p=0.0005; ****, p=0.00005.

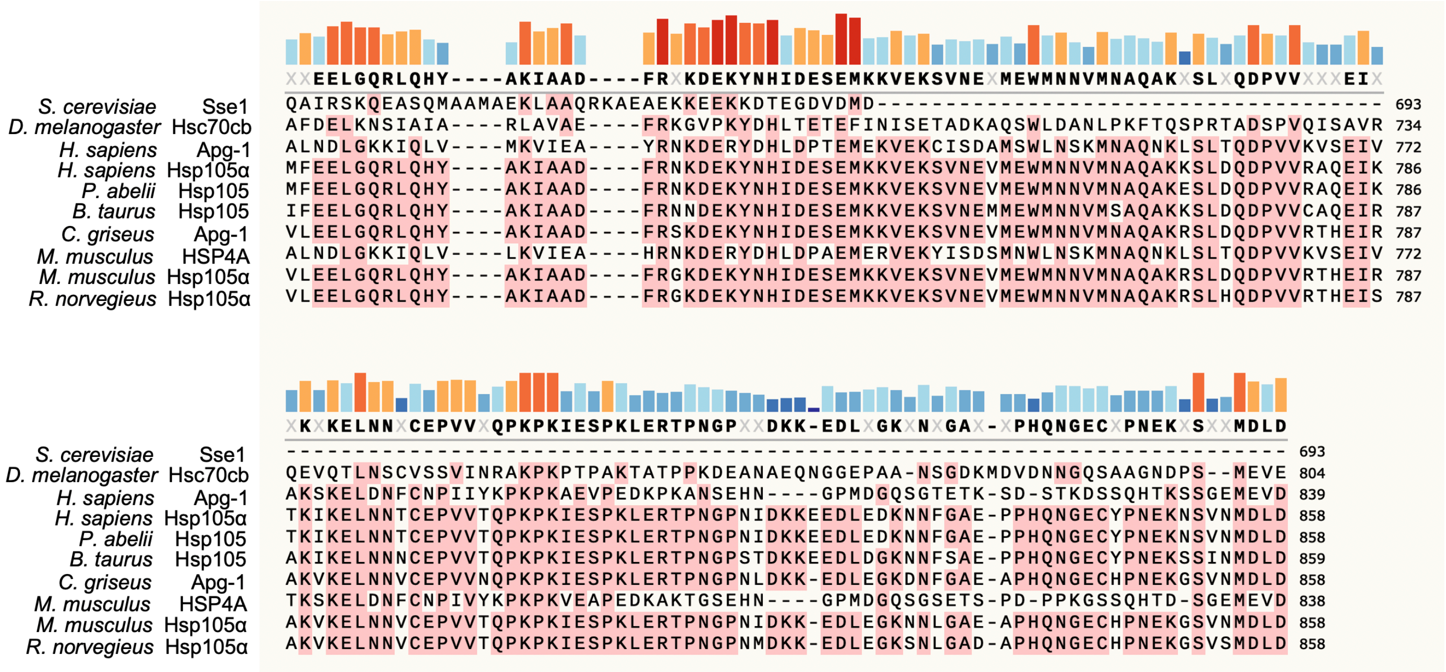

Figure S5: Multiple species alignment of Hsp110 carboxyl-terminus. SnapGene and Clustal Omega were used to align UniProt-verified sequences for Hsp110 homologs from the following organisms: budding yeast (*S. cerevisiae*), fruit fly (*D. melanogaster*), human (*H. sapiens*), orangutan (*P. abelii*), bovine (*B. taurus*), Chinese hamster (*C. griseus*), mouse (*M. musculus*), and rat (*R. norvegieus*). Consensus sequence indicated in **bold**. Residues included in the consensus sequence are present in more than 50% of aligned species. Amino acids matching the consensus sequence are highlighted in pink. Bars indicate percent conservation at a specific amino acid position. red: 75-100%; orange: 50-75%; blue <50%.

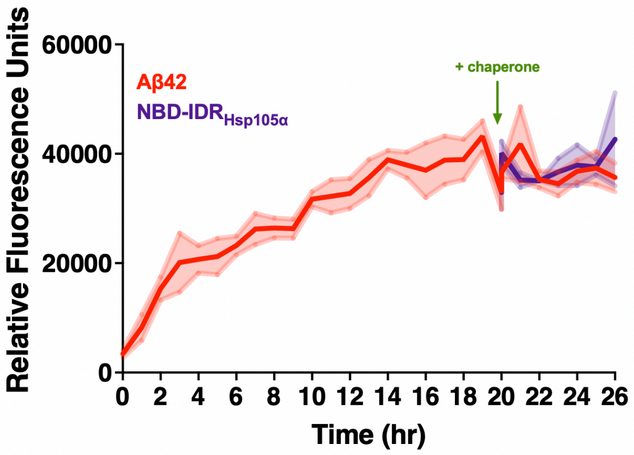

Figure S6: NBD-IDR$_{Hsp105\alpha}$ does not disassemble pre-formed Aβ42 fibrils. 2 µM Aβ42 was incubated alone for 20 hr as described in Materials and Methods, after which 4 µM NBD-IDR$_{Hsp105\alpha}$ was added to the reaction and allowed to incubate an additional 6 hr. Bolded lines are the average of three replicates for each condition while the shaded region represents standard error of the mean (SEM).

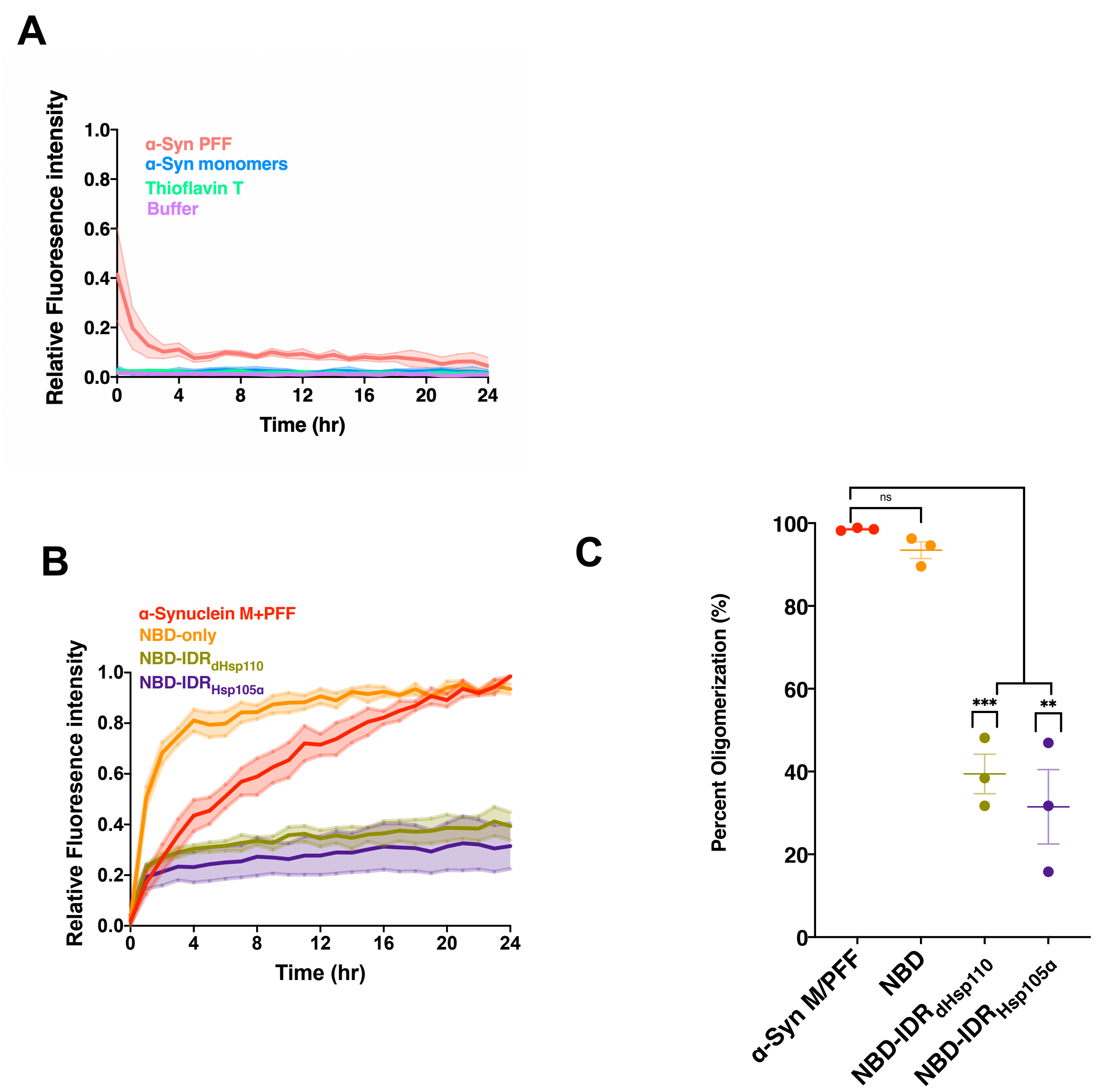

Figure S7: NBD-IDRs prevent α-synuclein oligomerization *in vitro*. A. Controls for thioflavin T binding assays and α-synuclein oligomerization. 2 μM α-synuclein monomers and 1 μM α-synuclein pre-formed fibrils were incubated individually in thioflavin T and Tris NaCl buffer. 5 μM thioflavin T was incubated alone in Tris NaCl buffer and fluorescence detected as described in Materials and Methods. Bolded lines are the average of three replicates for each condition while the shaded region represents standard error of the mean (SEM) **B.** 2 μM of α-synuclein monomers and 1 μM pre-formed fibrils incubated together (α-Synuclein M+PFF) or with 4 μM of respective chaperone: NBD, NBD-IDR$_{dHsp110}$, NBD-IDR$_{Hsp105α}$ were incubated for 24 hr and thioflavin T fluorescence detected as described in Materials and Methods. Bolded lines are the average of three replicates for each condition while the shaded region represents standard error of the mean (SEM). **C.** End point measurements of each condition were taken from S7B and divided by the α-Synuclein M+PFF measurement within the respective replicate and converted to relative percentage. Group differences were analyzed using Welch's *t*-test. *, p=0.05; **, p=0.005; ***, p=0.0005; ****, p=0.00005.

NBD-IDR$_{Hsp105\alpha}$

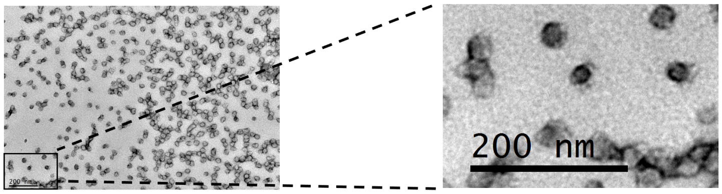

mean particle size: 33.6 nm +/- 2.6 nm

Figure S8: NBD-IDR$_{Hsp105\alpha}$ forms globular particles *in vitro*. 4 μM NBD-IDR$_{Hsp105\alpha}$ was incubated without substrate as described for Fig. 5 and imaged using transmission electron microscopy. Endpoint samples from the thioflavin T binding assay were recovered, negative stained and imaged via transmission electron microscopy, with or without additional chaperones as indicated. Particle size was measured (n=10) using ImageJ on the zoomed inset image. Scale bar= 200 nm.
